# Healthcare Quality Systems: International Frameworks, Evaluation and Improvement Strategies

**DOI:** 10.3390/healthcare14111510

**Published:** 2026-05-29

**Authors:** Christos Ntais, Michael A. Talias

**Affiliations:** Healthcare Management Program, School of Economics & Management, Open University of Cyprus, Nicosia 2220, Cyprus; christos.ntais@st.ouc.ac.cy

**Keywords:** healthcare quality, patient safety, accreditation, quality indicators, continuous quality improvement, patient-reported outcomes, quality management systems, healthcare delivery, digital health, health equity

## Abstract

Healthcare quality systems have evolved from narrow inspection and compliance mechanisms into broader, multi-level architectures that combine standards, measurement, organizational learning, patient safety, equity and patient-reported outcomes. Yet the field remains fragmented, with substantial variation in how quality is defined, measured and operationalized across countries and healthcare settings. This narrative review synthesizes major international quality systems and frameworks used in healthcare delivery, examines principal methods for evaluating and improving quality, and critically discusses organizational and policy conditions associated with successful implementation. A purposive review of the seminal conceptual literature and authoritative documents from major international organizations was undertaken to identify cross-cutting themes relevant to hospitals, ambulatory care and health systems. The review shows that influential approaches—including the World Health Organization’s quality and patient safety frameworks, Joint Commission International accreditation, NCQA/HEDIS, the EFQM model, ISO-based management systems, AHRQ quality indicators and OECD performance initiatives such as PaRIS—should be viewed as complementary rather than competing models. Their effectiveness depends less on formal adoption alone than on leadership commitment, workforce engagement, data infrastructure, patient involvement and alignment with financing and regulation. Evidence is strongest for gains in standardization, safety processes, teamwork and selected efficiency outcomes; direct causal effects on patient outcomes remain less consistent, particularly when quality systems become compliance-driven or are insufficiently adapted to local context. Future healthcare quality systems should integrate equity, digital interoperability, AI-enabled learning capabilities, patient-reported measures and continuous improvement while reducing measurement burden and indicator proliferation.

## 1. Introduction

Quality has evolved into an essential element of a healthcare system’s overall success. Early work by Donabedian established the enduring structure–process–outcome triad as a foundational framework for evaluating care, while the Institute of Medicine (IOM) broadened the field by defining high-quality care as safe, effective, patient-centered, timely, efficient and equitable [1,2]. Most recently, the World Health Organization (WHO) and the Lancet Global Health Commission on High-Quality Health Systems stressed that expanding access alone is insufficient if care is poorly coordinated, unsafe, disrespectful, or clinically ineffective [3,4].

Within this context, the quality systems in healthcare refer to the formal and informal arrangements by which organizations and health systems set standards, measure performance, assure accountability, promote improvement activities and learn from outcomes. Quality systems in healthcare now go far beyond just accreditation programs. They include indicator sets, external assessment mechanisms, patient safety initiatives, management standards, patient-reported measures, public reporting and continuous improvement methods embedded in everyday operations.

Despite widespread policy interest, the field remains conceptually and operationally heterogeneous. Different countries and organizations prioritize different goals—compliance, safety, efficiency, patient experience, equity, or organizational excellence—and often adopt several frameworks simultaneously. Therefore, healthcare leaders face a crowded quality landscape characterized by overlapping standards, limited comparative evidence, indicator proliferation and high reporting burden.

The aim of this narrative review is fourfold: (i) to synthesize major international quality systems and frameworks relevant to healthcare delivery; (ii) to describe the main methods used to assess and improve quality; (iii) to discuss organizational, digital and policy determinants that influence implementation; and (iv) to critically appraise what current evidence suggests about effectiveness and future directions. The intent of the review is interpretive rather than exhaustive. It is not designed to catalogue all national accreditation systems, disease-specific quality measures or local improvement programmes; instead, it provides an analytical synthesis for all stakeholders who require a unified perspective on contemporary quality systems in healthcare delivery.

The novelty of this review is its integrative focus. Rather than treating accreditation, quality indicators, organizational excellence models, patient-reported measures and continuous quality improvement as separate streams, the review analyses how these elements can be combined into a layered quality architecture. This perspective is particularly relevant for managers and policymakers who must align organizational governance, national policy requirements, data systems and patient-centered priorities within the same delivery system.

## 2. Materials and Methods

This study was conducted as a narrative review of the literature because the topic spans heterogeneous conceptual literature, empirical studies, policy reports and international standards rather than a single intervention or outcome suitable for meta-analysis. The narrative design was selected to support interpretive synthesis across organizational, policy and patient-centered dimensions of healthcare quality systems.

A targeted search of PubMed and Scopus was performed up to May 2026. Search strings combined terms related to quality systems and frameworks (“healthcare quality”, “quality systems”, “quality management”, “quality management system”, “accreditation”, “certification”, “external inspection”, “ISO 9001”, “ISO 7101”, “EFQM”, “Joint Commission International”, “NCQA”, “HEDIS”, “AHRQ quality indicators”, “OECD PaRIS”), measurement and improvement (“quality indicators”, “performance measurement”, “benchmarking”, “public reporting”, “clinical audit”, “continuous quality improvement”, “PDSA”, “Lean”, “Six Sigma”), and patient-centered domains (“patient safety”, “patient-reported outcome measures”, “PROMs”, “patient-reported experience measures”, “PREMs”, “safety culture”, “health equity”). Database searches were supplemented by manual reference-list screening and targeted review of documents from the WHO, Organisation for Economic Co-operation and Development (OECD), Agency for Healthcare Research and Quality (AHRQ), Joint Commission International (JCI), National Committee for Quality Assurance (NCQA), European Foundation for Quality Management (EFQM) and International Organization for Standardization (ISO).

Conceptual papers, systematic or scoping reviews, empirical studies and authoritative policy or standards documents were included if they addressed the definition, measurement, implementation or effectiveness of quality systems in healthcare delivery. Documents were excluded when they were narrowly disease-specific, limited to a local project without transferable lessons, focused primarily on biomedical mechanisms rather than healthcare delivery systems, superseded by newer policy guidance, or not available in English. No date restriction was applied, but priority was given to policy documents, standards, reviews and empirical studies published from 2018 onward to reflect current practice.

For transparency, the selection process was documented as a narrative-review screening log. The combined database, organizational website and citation-search process identified 312 potentially relevant records or documents. After duplicate removal and initial relevance screening, 147 sources were screened in title/abstract or document-summary form. Eighty-two were excluded because they were disease-specific, too local in scope, unrelated to healthcare delivery quality systems, or superseded. Sixty-five full texts or full documents were assessed. Nineteen were excluded after full-text assessment because of narrow specialty focus (n = 7), insufficient relevance to quality system implementation or evaluation (n = 6), duplication of superseded guidance (n = 4), or lack of accessible detail (n = 2). Forty-six sources were retained for the final narrative synthesis.

The selected literature was synthesized narratively and organized around the main themes of the review: conceptual foundations, major international quality frameworks, methods for assessing and improving quality, determinants of implementation, evidence on effectiveness and future directions. No formal study-level risk-of-bias assessment was undertaken. This decision is consistent with the narrative purpose of the review, which includes policy and standards documents that are not amenable to conventional appraisal tools. Nevertheless, the absence of structured critical appraisal is a limitation: the review should not be interpreted as estimating pooled effect sizes or proving causal effects of any framework.

## 3. Conceptual Foundations of Quality in Healthcare

Three conceptual constructs continue to guide modern thought regarding healthcare quality. The first is Donabedian’s distinction between structure, process and outcomes [1]. Specifically, structural components comprise facilities, staffing, equipment, governance and information systems; process measures reflect the actual activities performed by clinicians and organizations; and outcome measures reflect the effects of the care delivered relative to mortality, morbidity, functionality, experience and quality of life. While simplistic, this model remains analytically powerful due to its recognition that failure to deliver quality care may arise from deficient inputs, unreliable care processes, or poor outcomes despite apparently adequate structures.

The second construct is the IOM’s multi-dimensional definition of quality [2]. The six aims—safety; effectiveness; patient-centeredness; timeliness; efficiency; and equity—highlight the trade-offs inherent in healthcare delivery. A service may be technically effective yet inequitable; efficient yet insufficiently patient-centered; or timely for some populations but not for others.

The third conceptual construct is the system-level and people-centered framing advanced by WHO and the high-quality health systems literature [3,4,5,6]. The WHO defines quality as the degree to which health services increase the probability of achieving desired health outcomes and are consistent with current professional knowledge [3]. The WHO also highlights that quality should be effective, safe, people-centered, timely, equitable, integrated and efficient [3,5,6]. This broader formulation is especially relevant to contemporary delivery systems, where patients often traverse multiple providers, settings and technologies. In such environments, fragmentation itself becomes a quality problem.

These conceptual models indicate that quality systems should not be reduced to compliance with standards. A mature system connects governance, measurement, workforce behavior, safety culture, patient experience, equity and learning over time. It also recognizes that trust and fairness are not peripheral values but core components of high-performing health systems [4,5,6,7].

## 4. Major International Quality Systems and Frameworks

International quality systems vary in scope, level of application and underlying theory of change (Table 1). Some are policy frameworks, some are external accountability or accreditation systems, and others are measurement, management or continuous improvement models. A structured comparison is useful because many organizations adopt several of these approaches simultaneously, often without clearly distinguishing their purposes.

The WHO has played a central role in shifting quality from an institutional concern to a national policy priority. Its Handbook for National Quality Policy and Strategy provides countries with a structured approach for establishing governance, stakeholder engagement, national aims, measurement and improvement capacity [5]. This quality agenda is closely linked to universal health coverage (UHC): coverage expansion cannot achieve its intended population-health benefit if services are unsafe, ineffective, disrespectful or poorly coordinated [3,4,5]. The WHO’s patient safety agenda deepens this quality–UHC connection. The Global Patient Safety Action Plan 2021–2030 frames avoidable harm as a system problem that requires policy action, organizational learning, patient and family engagement, safe clinical processes and reliable reporting [6]. The Global Patient Safety Report 2024 shows that progress remains uneven across countries and highlights the need for sustained investment in institutional capacity, measurement and implementation support [7].

Joint Commission International (JCI) has emerged as one of the most prominent global accreditation systems, particularly in hospital care [8]. Its standards emphasize patient-centered processes, medication management, infection prevention and control, governance, workforce competence and facility safety. JCI’s strength lies in its ability to assess an entire organization comprehensively and establish a common language of quality and safety across jurisdictions. Its limitation is that accreditation can become sporadic, documentation-driven or survey-oriented if it is not embedded in broader organizational learning.

In the US context, the National Committee for Quality Assurance (NCQA) and the Healthcare Effectiveness Data and Information Set (HEDIS) have driven ambulatory and payer-oriented quality measurement at scale [9]. HEDIS operationalizes quality improvement through standardized indicators that support benchmarking, contracting and public accountability. Compared with accreditation, this approach is more explicitly data-driven and population-oriented; however, it may underestimate relational, experiential and contextual aspects of care when used without complementary qualitative or patient-reported information.

The European Foundation for Quality Management (EFQM) offers an organizational excellence perspective [10]. Unlike clinical compliance-based accreditation models, EFQM addresses leadership, strategy, people, partnerships, resources, processes and results. This makes it attractive for healthcare organizations seeking integration between quality, strategy and performance management, although it is broader and less explicitly clinical than some healthcare-specific accreditation models.

International Organization for Standardization (ISO)-based approaches occupy a distinct position in the quality. ISO 9001 is a general quality management systems standard based on process control, documentation, continual improvement and customer focus [11]. The more recent ISO 7101 standard is specifically designed for quality management systems in healthcare organizations [12]. Together, these standards signal a transition from generic quality management toward sector-specific quality architectures, although implementation experience with ISO 7101 remains limited.

Indicator-based systems are also fundamental. The Agency for Healthcare Research and Quality (AHRQ) quality indicators use routinely collected data to track clinical performance and outcomes [13]. Internationally, the OECD Health Care quality indicators (HCQI) programme and Health at a Glance reports support cross-national comparison, while OECD PaRIS expands assessment into patient-reported domains, especially for people living with chronic conditions in primary care [14,15]. These systems shift quality from internal self-assessment toward comparative performance intelligence, but they require careful interpretation because coding practices, case mix and health-system context differ.

**Table 1:** Major international quality systems and frameworks relevant to healthcare delivery.

Framework/System	Primary Level of Application	Main Orientation	Typical Strengths	Common Limitations or Caveats
WHO Quality, Patient Safety and UHC ^1^ Frameworks	National and health-system level	Policy architecture linking quality, safety, people-centered care and UHC	Supports national governance, stakeholder engagement, safety priorities and system-wide measurement	Requires political commitment, implementation capacity, financing and adaptation to local context
JCI Accreditation	Healthcare organization/hospital	External standards, survey and accreditation	Creates a common safety language and strengthens governance, infection prevention, medication safety and facility processes	Can become episodic, documentation-driven or survey-focused if not linked to daily learning
NCQA/HEDIS	Payer, ambulatory and population-health level	Standardized quality measurement, benchmarking and accountability	Scalable indicators for contracting, public accountability and population management	May underrepresent relational, experiential and contextual aspects of care; primarily US-oriented
EFQM Model	Organization/enterprise level	Organizational excellence, leadership, strategy, results	Integrates quality with management, transformation and long-term performance	Less clinically specific than healthcare-focused accreditation systems
ISO 9001 and ISO 7101	Organization/quality management system	Process standardization, documentation, risk-based thinking and continuous improvement	Formalizes workflows, corrective action and healthcare-specific quality governance	Generic ISO 9001 is not clinical [11]; ISO 7101 diffusion and implementation evidence remain early [12]
AHRQ Quality Indicators	Hospital/system analytics	Administrative data-based quality and safety measurement	Feasible, standardized and useful for surveillance and benchmarking	Dependent on coding quality, may miss clinical nuance or patient experience
OECD HCQI/PaRIS	Cross-national health system comparison	Comparative performance measurement and patient-reported outcomes/experiences	Enables international benchmarking and inclusion of patient voice	Cross-country comparability and contextual interpretation remain challenging

^1^ UHC: universal health coverage.

**Figure 1:**
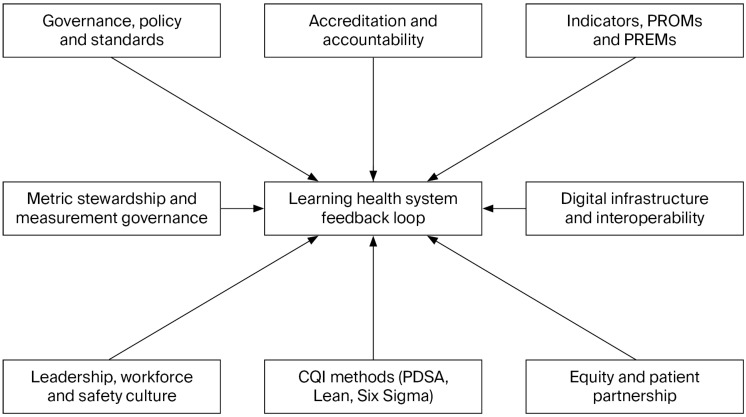
Integrated, layered quality architecture linking governance, standards, accreditation, measurement, digital infrastructure, patient partnership, workforce culture and continuous quality improvement.

Taken together, these frameworks should be viewed as complementary. Accreditation can strengthen structures and accountability, indicator systems can monitor variation; management standards can align strategy and processes; and patient-reported systems can reveal domains of care missing from administrative data. The practical challenge is not to select one universal “best” system, but to design an integrated portfolio suited to organizational purposes, policy context, data maturity and patient needs. Figure 1 presents this layered architecture.

## 5. Methods for Assessing and Improving Quality in Healthcare

Measurement is central to all quality systems, but measurement alone does not improve care. High-performing organizations combine assessment methods that diagnose quality problems with improvement methods that redesign processes and close feedback loops (Table 2).

Quality indicators remain the most commonly used tools. They can be either structural (e.g., staffing ratios or availability of electronic prescribing), process-based (e.g., adherence to sepsis bundles or vaccination coverage), outcome-based (e.g., mortality, readmissions or infection rates), or patient-reported (e.g., symptom burden, functional status or patient experience) [1,16,17]. A strong indicator set should be valid, feasible, interpretable, responsive to change and balanced across domains to prevent tunnel vision [16,17]. Overly narrow portfolios of indicators can encourage gaming, selective documentation or improvement in measured domains at the expense of unmeasured priorities.

Practical metric stewardship is therefore essential. Organizations should maintain an inventory of required measures, map overlap across regulators and payers, retire low-value indicators, prioritize a small set of high-impact measures, harmonize definitions, automate extraction where possible and ensure that dashboards provide actionable feedback to frontline teams. Recent efforts to align measures across programmes illustrate the value of parsimonious, broadly applicable and digitally reportable measure sets [18,19].

Clinical audit and feedback are core methods for assessing quality. Clinical audit compares current practices with an explicit standard, while feedback communicates performance data to teams or providers who can act on it. Audit is most useful when the target practice is evidence-based but inconsistently implemented. Its effectiveness depends on timely data, credible measurement, psychologically safe feedback and whether recipients have the authority and resources to change practice.

External inspection, accreditation and certification assess whether organizations meet defined standards. These approaches can improve attention to governance, documentation, standardization and safety systems, but evidence remains mixed regarding direct effects on patient outcomes [20,21,22,23]. Their value is greatest when survey findings are translated into routine improvement work, board-level oversight and learning cycles rather than treated as one-time evidence of compliance.

Benchmarking and public reporting allow organizations to compare themselves with peer groups, high performers, or national averages. Benchmarking can identify outliers and strategic priorities, but it is useful only when indicators are comparable, risk adjustment is credible and organizations have analytic capacity to interpret variation [14,17]. Without these conditions, benchmarking may create reputational pressure, encourage risk avoidance or lead to defensive documentation rather than improvement.

PROMs and PREMs are increasingly important because they capture domains that administrative and clinical metrics often miss: symptom burden, functioning, treatment burden, communication quality, coordination, trust and perceived benefit of care [15,24,25]. Operationally, implementation requires careful instrument selection, cultural and language adaptation, decisions about collection mode and timing, case-mix adjustment, management of missing data and response bias, and clear thresholds for clinical action. Their value is maximized when data are embedded in clinical pathways, shared decision-making, patient portals, multidisciplinary review and organizational learning rather than collected only for symbolic reporting [24,25,26,27].

Patient safety culture assessments provide insight into teamwork, communication openness, non-punitive responses to error, staffing adequacy and leadership support for safety [28,29,30,31]. Although safety culture surveys do not measure safety directly, they reveal the social and organizational conditions within which safe care is created or compromised. Their results should therefore be paired with concrete improvement work, leadership development and follow-up measurement.

Continuous quality improvement (CQI) methods, especially Plan–Do–Study–Act (PDCA) cycles, Lean, Six Sigma and Lean Six Sigma, translate measurement into process redesign [32,33,34,35]. These methods are most effective when approached as a management discipline rather than as isolated projects. Their advantages include reducing delays, waste, defects and unwanted variation. Their risks include superficial tool adoption, work intensification and failure to address clinical priorities or frontline realities.

The learning health system concept provides a bridge between measurement and CQI. In a learning system, routinely collected clinical, administrative and patient-reported data are combined with external evidence, interpreted through feedback loops and used to adapt care processes in near real time [36,37]. This makes learning health systems a useful organizing concept for modern quality architectures because they integrate digital infrastructure, evidence use, implementation science, patient partnership and continuous improvement.

## 6. Determinants of Effective Implementation

The success of a quality system depends as much on implementation context as on technical design. Quality initiatives that appear sound on paper often fail when organizations lack absorptive capacity, data capability, leadership attention or workforce trust [32,38]. Implementation should therefore be understood as an organizational change process, not simply as adoption of a framework.

Leadership and governance are first-order determinants. Quality systems are more likely to succeed when senior leaders frame quality as a strategic priority, model transparency, allocate resources, review meaningful data and maintain continuity beyond accreditation or reporting cycles [5,6,30,38]. Empirical reviews of accreditation and CQI repeatedly show that benefits are mediated by professional engagement, resources and integration into routine work [21,22,32]. Conversely, episodic leadership attention can convert quality programmes into short-term campaigns that fade once external pressure decreases.

Workforce capability and engagement are equally important. Quality systems are implemented by clinicians, managers, quality officers and support staff. Staff need clear roles, training in data interpretation and improvement methods, time to participate and confidence that measurement is used for learning rather than blame [28,32,34,35,38]. Practical examples include multidisciplinary review of infection-control data, medication-safety huddles, audit-and-feedback cycles for care bundles and Lean redesign of patient flow. In each case, data alone are insufficient unless frontline teams can interpret results and change work processes.

Organizational culture shapes whether quality systems become routine practice or ceremonial structures. Teamwork, interprofessional communication and psychological safety determine whether staff report hazards, discuss variation and experiment with improvement. A hospital may have excellent protocols on paper yet still generate preventable harm if staff fear blame, if professional groups work in silos, or if feedback loops are slow and opaque.

Digital maturity is a decisive enabling factor. Modern quality systems depend on reliable coding, interoperable electronic health records (EHR), registries, timely dashboards and the ability to link clinical, administrative and patient-reported data [13,15,24,25,39,40,41]. Interoperability allows quality information to follow patients across settings and reduces duplicate data entry. Poorly designed systems, however, can increase documentation burden, produce misleading measures or fragment workflows. Digital transformation should therefore be judged by whether it makes care safer, more coordinated and more usable for clinicians and patients.

Regulatory environments, payment models and incentives also influence implementation. Value-based payment, public reporting and accreditation requirements can focus attention on quality, but poorly aligned incentives may encourage selective documentation, avoidance of high-risk patients, or concentration on measured rather than meaningful outcomes. Policy design should reward learning, equity-sensitive improvement and sustained capability rather than simple target attainment.

Patient engagement and equity should be treated as implementation determinants rather than optional additions. Quality systems that ignore health literacy, language, cultural expectations, disability, geography and financial barriers may improve average performance while widening disparities. Similarly, systems that fail to involve patients in selecting, interpreting and acting on measures may overlook dimensions of quality that matter most to people receiving care [3,4,5,6,7,15,24,25].

Implementation challenges are amplified in low- and middle-income countries. Quality systems may have to operate with infrastructure deficits, workforce shortages, limited laboratory or information systems, fragmented supply chains and multiple donor or regulatory reporting requirements. In such contexts, international frameworks should be phased, adapted and prioritized around essential safety, respectful care, reliable processes and a small number of locally actionable indicators rather than imported as resource-intensive compliance packages [4,5,6,7].

## 7. What Does the Evidence Show About Effectiveness?

The current body of evidence suggests that quality systems can improve healthcare delivery, but effects vary by framework, context and implementation depth (Table 3). Benefits are most consistently observed in organizational processes, standardization, documentation quality, teamwork and selected operational outcomes. Direct, causal effects on patient outcomes are less consistently demonstrated.

For accreditation, the literature is cautiously supportive but not unequivocal. Systematic reviews report plausible benefits in organizational performance, patient safety practices and compliance with standards, yet they emphasize heterogeneity in study design, setting and outcomes [20,21,22,23,42]. A Cochrane review of external inspection found the certainty of evidence to be very low, highlighting the scarcity of high-quality controlled studies [20]. Recent reviews suggest that accreditation can stimulate quality improvement, but its benefits are mediated by professional engagement, resources and integration into routine work [21,22].

Evidence on ISO and EFQM in hospitals is more limited and largely observational. Available reviews indicate potential benefits related to standardization, patient satisfaction, selected efficiency indicators and performance management, but the underlying studies are heterogeneous and vulnerable to confounding [43]. These approaches appear most useful when organizations seek to institutionalize process discipline and enterprise-wide quality governance rather than when they are used as stand-alone clinical interventions.

CQI methods show more consistent operational gains. A recent scoping review found that quality improvement approaches can support better process reliability, stronger teamwork and organizational learning [32]. Lean-oriented reviews likewise show potential gains in throughput, waiting times, workflow efficiency and selected quality and satisfaction outcomes, although effect sizes vary and negative side effects—including work intensification or superficial tool adoption—remain possible [33,34,35].

Studies of PROM/PREM-based strategies present a nuanced picture. The strongest evidence exists at the clinical level, particularly when PROMs are used to monitor symptoms, trigger care pathways, or support decision-making [24,26,27]. Benchmarking-only uses show weaker and less consistent effects [26]. This suggests that patient-reported data are most impactful when they are actionable within care settings rather than used solely as external performance metrics.

Evidence on safety culture interventions is expanding. Systematic reviews indicate that culture-focused interventions may improve leadership, teamwork, recognition of stress, job satisfaction and workplace conditions and may reduce burnout-related outcomes among staff [28,29]. Leadership style is important; transformational and ethical styles appear particularly relevant for developing safety culture [30]. The relationship between safety culture and patient experience remains less clear, and mixed-methods research is needed to clarify causal mechanisms [31].

Overall, the evidence supports a practical conclusion: quality systems are most effective when implemented as interconnected socio-technical strategies rather than isolated instruments. A hospital can receive accreditation yet fail to improve continuously; it can collect PROMs yet fail to use them clinically; and it can publish dashboards yet fail to learn from variation. Sustainable success depends on integration.

This distinction is especially important when comparing accreditation-driven systems with CQI models. Accreditation usually emphasizes external standards, periodic assessment and accountability. CQI emphasizes local problem-solving, rapid feedback and iterative redesign. Accreditation can define the floor for safe and reliable care, while CQI can help organizations move beyond minimum compliance toward adaptive learning. The risk of accreditation is bureaucratic ritual; the risk of CQI is fragmented local experimentation without governance. Mature quality architectures combine both.

## 8. Persistent Challenges and Future Directions

Several persistent challenges limit the positive effect of quality systems. First, indicator proliferation has created substantial measurement burden. Organizations often report to multiple agencies using overlapping but non-identical definitions, consuming analytic and clinical resources while weakening coherence. High-value measurement requires fewer, better and more actionable indicators rather than endless expansion [16,17,19,36,39].

Administrative costs and bureaucratic risks should be explicitly recognized. Accreditation preparation, consultant fees, survey readiness activities, data abstraction, documentation audits and parallel reporting systems can divert time from patient care and improvement. Bureaucratic risk arises when staff focus on producing evidence of compliance rather than improving care processes. This risk is not an argument against quality systems; it is an argument for proportionate requirements, harmonized indicators, automated data capture and governance that asks whether each measure or standard adds practical value.

Second, the evidence base remains methodologically uneven. Many studies evaluate quality improvement efforts using before-and-after designs that are vulnerable to secular trends and selection bias. This is particularly important for accreditation and certification, because stronger organizations may be more likely to pursue accreditation and to perform well regardless [20,21,22,23,42].

Third, context sensitivity remains underdeveloped. Interventions that work in large tertiary hospitals may not transfer directly to primary care, community services, low-resource settings or fragmented provider markets. International frameworks should therefore be adapted rather than transplanted. In low- and middle-income settings, quality systems must balance ambition with feasibility by prioritizing essential safety processes, respectful care, workforce capacity, reliable supplies, basic information systems and a small set of locally actionable indicators [4,5,6,7].

Fourth, equity must become a design principle rather than a reporting add-on. Quality indicators should be stratified by socioeconomic status, geography, migration status, disability, gender, age and other locally relevant dimensions of disadvantage. Without stratification, improvements in average performance may mask unequal benefit. Equity-sensitive quality systems also require community engagement, culturally appropriate PROM/PREM tools and attention to digital exclusion.

Fifth, digital transformation creates opportunities and risks. Interoperable EHR, registries, automated extraction, real-time dashboards, clinical decision support and advanced analytics can make quality measurement more timely and less burdensome [40,41]. Artificial intelligence may support risk prediction, anomaly detection, documentation support and prioritization of improvement opportunities, but it also introduces risks related to bias, explainability, privacy, cybersecurity and accountability [44]. Digital maturity should therefore be evaluated not only by technical capacity but by whether data systems improve safety, coordination, usability and equity.

An encouraging future direction is the emergence of integrated and learning-oriented quality systems. ISO 7101 indicates movement toward healthcare-specific quality management systems [45], while OECD PaRIS expands the ability of health systems to measure what patients report about outcomes and experiences across countries [46]. The updated AHRQ framework for national quality and disparities reporting recognizes that performance is multi-faceted and should include quality and disparity domains rather than a few stand-alone metrics [39]. Learning health system models extend this logic by connecting data, evidence, implementation and feedback loops so that care delivery becomes continuously self-improving [36,37].

Over time, the most effective quality systems are likely to be those that combine governance, digital interoperability, patient-reported data, safety culture, equity stratification and CQI in a single learning architecture. The future challenge is not simply to add more measures or standards, but to make quality systems more usable, less burdensome and more responsive to patient and population needs.

## 9. Conclusions

Quality systems in healthcare delivery have evolved into a broad set of standards, metrics, governance tools, patient safety strategies, patient-reported measures and learning methods. No single system provides a complete solution. Accreditation can support accountability and standardization; indicator systems can reveal variation; management standards can strengthen organizational discipline; PROMs and PREMs can restore the patient voice; digital infrastructure can enable timely feedback; and CQI methods can translate data into redesigned care processes.

The central lesson of this literature review is that quality improvement is not secured by formal adoption alone. The effectiveness of quality systems relies on leadership, organizational culture, workforce engagement, digital capability, patient partnership, equity-sensitive measurement and alignment with regulation and financing. When these conditions are absent, quality systems risk becoming symbolic, administratively burdensome or excessively compliance-driven.

For healthcare organizations and policymakers, the practical implication is clear: quality should be viewed as a multi-layered architecture rather than a single programme. Future quality systems should be strategically governed, empirically measured, digitally enabled, equity-sensitive, patient-centered and continuously learning.

## Figures and Tables

**Table 2:** Common methods used to assess and improve quality in healthcare delivery.

Method	Typical Data/Metrics	Best Use	Main Advantages	Key Limitations
Structure, process and outcome indicators	Staffing, compliance, mortality, readmissions, infections, experience, PROMs	Monitoring performance over time and across units	Quantifiable and comparable; supports accountability and improvement	Risk of tunnel vision, gaming, overmeasurement or inequitable improvement
Clinical audit and feedback	Chart review, adherence to protocols, care bundles	Improving reliability of defined practices	Actionable and clinically focused	Effect depends on data timeliness, credibility and local authority to change practice
Accreditation/certification/external inspection	Standards compliance, survey findings, corrective actions	Strengthening governance, standardization and safety systems	Creates organizational focus and external accountability	Causal link to patient outcomes is often difficult to demonstrate; may create documentation burden
Benchmarking and public reporting	Peer comparison dashboards, national reports, league tables	Identifying outliers and strategic priorities	Encourages transparency and comparative learning	Requires risk adjustment and analytic capability; may create reputational pressure without learning
PROMs and PREMs	Symptoms, functioning, communication, trust, coordination, experience	Patient-centered evaluation, shared decisions and pathway redesign	Captures domains invisible to administrative data	Requires validated tools, workflow integration, case-mix interpretation and action on feedback
Safety culture assessment	Survey-based measures of teamwork, openness, staffing, leadership	Diagnosing organizational conditions for safe care	Illuminates social and cultural barriers to improvement	Measures perceptions rather than safety events directly
CQI ^1^ methods (PDSA, Lean, Six Sigma, Lean Six Sigma)	Cycle times, defects, variation, delays, handoffs	Redesigning processes and reducing waste or error	Translates measurement into operational change	Fails when treated as a short-term toolkit rather than a management system
Digital quality infrastructure	EHR ^2^, registries, interoperability, automated extraction, real-time dashboards	Enabling timely measurement and feedback	Reduces manual reporting and supports learning	Requires data quality, privacy, cybersecurity, usability and digital inclusion
Learning health system feedback loops	Routine data, evidence, patient input, implementation feedback	Connecting measurement to continuous learning	Integrates analytics, CQI, evidence and patient partnership	Requires leadership, governance, analytic capacity and sustained investment

^1^ CQI: continuous quality improvement; ^2^ EHR: electronic health records.

**Table 3:** Interpreting the evidence base for major quality system approaches.

Approach	Most Consistent Benefits Reported	What Remains Uncertain	Implementation Message
Accreditation/external standards	Better standardization, documentation, governance attention and some safety processes	Magnitude of effect on patient outcomes and cost-effectiveness	Use accreditation as a platform for learning, not as an end in itself
ISO/EFQM-based management systems	Improved process discipline, strategic alignment and selected performance indicators	Generalizability across settings, independent clinical effect	Most useful where organizations need stronger system-wide quality governance
Lean/CQI/Lean Six Sigma	Shorter waits, improved flow, less waste, stronger process reliability and some satisfaction gains	Durability of gains and risk of work intensification	Frontline ownership and local data are essential
PROM/PREM programmes	Better symptom monitoring, communication, shared decisions and patient-centered pathway improvement	Best large-scale implementation and benchmarking models	The closer the data are to care decisions, the greater the likely value
Safety culture interventions	Improved teamwork, communication, stress recognition and selected staff outcomes	Direct causal pathways to patient outcomes	Culture work should be paired with leadership development and process redesign
Learning health systems	Improved feedback loops, evidence uptake, data-informed redesign and spread of local learning	How to evaluate maturity, scalability and equity impact	Requires aligned governance, digital infrastructure, analytic capacity and improvement execution

## Data Availability

No new data were created or analyzed in this study.
